# Prognostic risk factor of major salivary gland carcinomas and survival prediction model based on random survival forests

**DOI:** 10.1002/cam4.5801

**Published:** 2023-03-19

**Authors:** Yufan Chen, Guoli Li, Wenmei Jiang, Rong Cheng Nie, Honghao Deng, Yingle Chen, Hao Li, Yanfeng Chen

**Affiliations:** ^1^ State Key Laboratory of Oncology in South China Guangzhou China; ^2^ Collaborative Innovation Center for Cancer Medicine Guangzhou China; ^3^ Endoscope Center Sun Yat‐sen University Cancer Center Guangzhou China; ^4^ Department of Head and Neck Surgery Sun Yat‐sen University Cancer Center Guangzhou China

**Keywords:** machine learning, major salivary gland tumors, prediction model, prognosis, random survival forest

## Abstract

Salivary gland malignancies are rare and are often acompanied by poor prognoses. So, identifying the populations with risk factors and timely intervention to avoid disease progression is significant. This study provides an effective prediction model to screen the target patients and is helpful to construct a cost‐effective follow‐up strategy. We enrolled 249 patients diagnosed with salivary gland tumors and analyzed prognostic risk factors using Cox proportional hazard univariable and multivariable regression models. The patients' data were split into training and validation sets on a 7:3 ratio, and the random survival forest (RSF) model was established using the training sets and validated using the validation sets. The maximally selected rank statistics method was used to determine a cut point value corresponding to the most significant relation with survival. Univariable Cox regression suggested age, smoking, alcohol consumption, untreated, neural invasion, capsular invasion, skin invasion, tumors larger than 4 cm, advanced T and N stage, distant metastasis, and non‐mucous cell carcinoma were risk factors for poor prognosis, and multivariable analysis suggested that female, aging, smoking, untreated, and non‐mucous cell carcinoma were risk factors. The time‐dependent ROC curve showed the AUC of the RSF prediction model on 1‐, 2‐, and 3‐year survival were 0.696, 0.779, and 0.765 respectively in the validation sets. Log‐rank tests suggested that the cut point 7.42 risk score calculated from the RSF was most effective in dividing patients with significantly different prognoses. The prediction model based on the RSF could effectively screen patients with poor prognoses.

## Graphical abstract

Random survival forest(RSF) is a quite new algorithm based on machine learning in survival analysis. The model applying the RSF in the present study helps us determine the risk of patients at a specific time point, so the strategy of follow‐up could be more cost‐effective. And patients at high risk could also be screened by calculating the risk score.

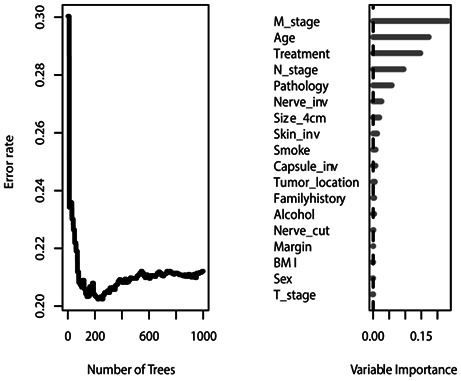

## 1 INTRODUCTION

Carcinomas of the salivary glands are heterogeneous and rare. It takes up less than 1% of malignant head and neck neoplasms.^1^, ^2^, ^3^, ^4^ The histopathological classification of malignant gland tumors is complex,^5^ and it is tough for surgeons and pathologists to determine its origin.

The carcinomas of the major salivary glands (C‐MSG) occur in three sets of paired glands: the parotid, the submandibular, and the sublingual gland. The parotid is the most common site of onset, while tumors derived from the sublingual gland are usually malignant.^6^ Due to their rarity and histopathologic variety, prognostic judgment was challenging. There is also an urgent need for proper and effective treatments to control the tumor and reduce facial nerve injury.^7^ Furthermore, knowledge of the risk factors is significant for reasonable follow‐up strategies to prevent tumor recurrence.^8^

Clinical prediction models are extensively used in current studies, and proper selection of the model is the cornerstone.^9^, ^10^ The Cox model is the most widely used,^11^ associated with cancer survival, and identifies risk factors effectively with a respectively straightforward procedure. However, with electronic patient files bringing much more information, an easily automated model is needed. The random survival forest (RSF) is a good example. It does not have to satisfy strict assumptions like the Cox model, which widens its use, and performs better in some ways.^12^, ^13^, ^14^, ^15^ Breiman first introduced RSF in 2001,^16^ and the specified package “randomForestSRC”^17^ of R software is developed to apply better use of the model and augment visualization of the results.^18^

This retrospective analysis was undertaken to identify prognostic factors for the overall survival (OS) of patients with salivary gland tumors using the Cox model. For better prediction of the prognosis, the RFS model was performed to screen patients at high risk.

## 2 MATERIALS AND METHODS

### 2.1 Patient selection

The clinical data of 249 patients with histologically confirmed C‐MSG treated at Sun Yat‐sen University Cancer Center between January 2000 and December 2013 were included in the study.

### 2.2 Demographic and clinical variables

Information on demographic features and clinical–pathological features, including regional invasion, recurrence, treatment modality, and other vital information, was extracted from the hospital information system (HIS) database. Tobacco use and alcohol assumption were recorded as well. Body mass index (BMI) less than 25 was clarified as normal, and the others were overweight. Tumor size and the presence of cervical lymph node metastases were evaluated through various imaging examinations, including CT, MRI scans, and ultrasound. The presence of distant metastasis was assessed by X‐rays or CT, bone scintigraphy, ultrasound, or PET‐CT examinations. All cases were staged according to the WHO Classification for Tumors (2005) and the AJCC TNM Staging System (8th edition) for salivary gland tumors.^5^, ^19^

### 2.3 Follow‐up

Information on the 249 patients with C‐MSG was collected by letter, telephone, and outpatient follow‐up visits. Follow‐up continued until December 2018. OS was calculated from the date of definitive diagnosis to the date of death.

### 2.4 Statistical analysis

**FIGURE 1:**
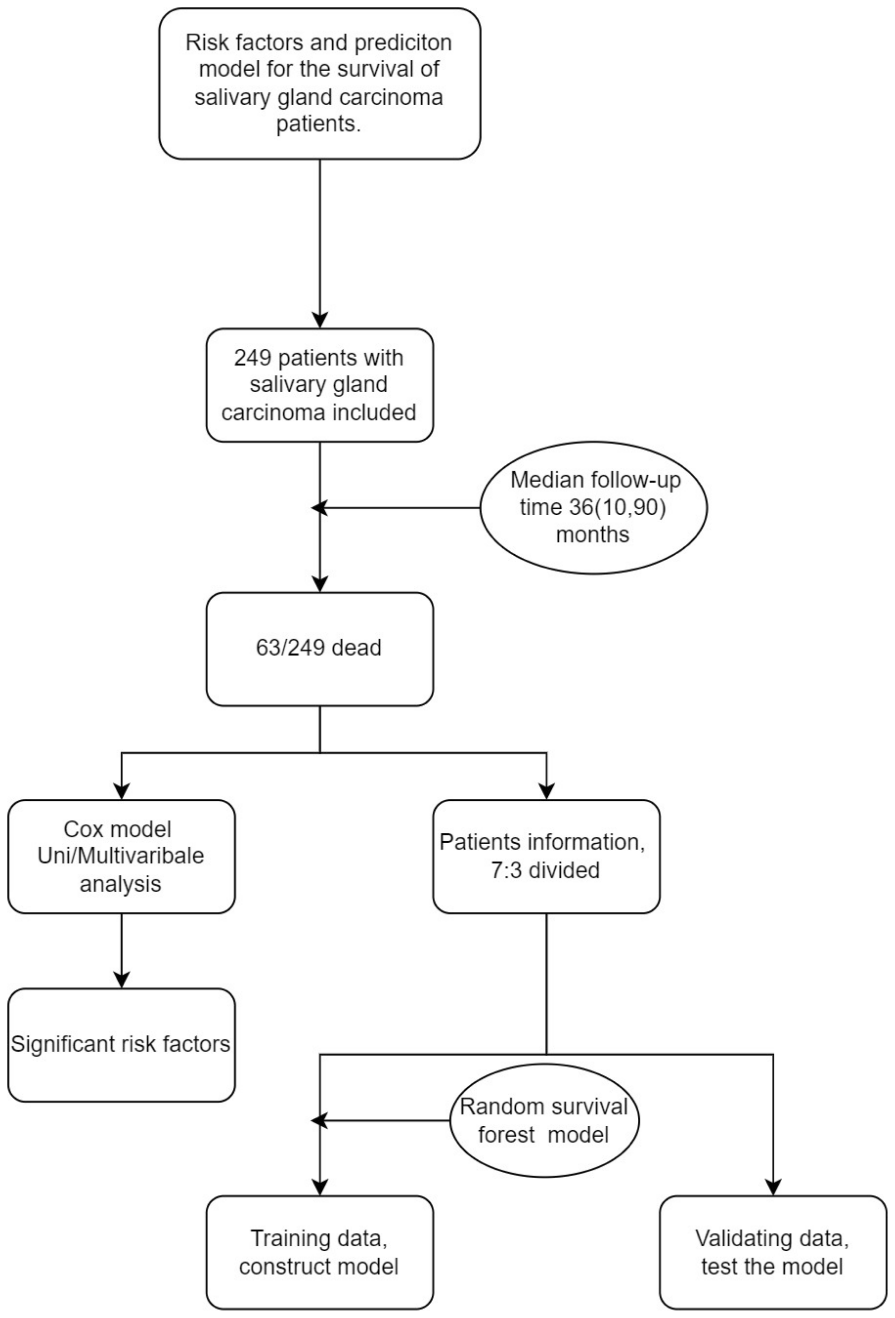
Flow diagram of the present study.

Continuous variables were summarized as mean ± standard deviation. Categorical variables were presented as frequency (percentage). Univariable and multivariable Cox proportional hazards regression analyses were performed to determine the risk factors. Hazards ratio (HR) was presented with its 95% confidence intervals (CI) as HR (low CI, high CI), and HR less than 1.0 was regarded as a protective factor. *p* < 0.05 suggested statistical significance. Data of the patients were split into training and validation sets in a 7:3 ratio, and the survival prediction models were developed using RSF in the training set. Time‐dependent receiver operator characteristic curve (ROC) analysis^20^, ^21^ was performed to evaluate the accuracy of the prediction model. The maximally selected rank statistics method was used to determine a value of a cut point that corresponds to the most significant relation with survival. Kaplan–Meier method was used to estimate the survival distributions, and the log‐rank test was performed for the comparison of OS. Figure 1 shows the flow diagram of our study. Data processing was carried out using R version 4.1.3.

## 3 RESULTS

### 3.1 Patient characteristics

The characteristics of the 249 patients who satisfied the criteria above in this study are summarized in Table 1. Sixty‐three patients (25.3%) died during the follow‐up from 0 to 165 months (median, 36.0 months). The 3 and 5‐year O.S. were 80.7% and 77.1%, respectively.

**TABLE 1:** Characteristics of patients and Cox univariable and multivariable survival analysis.

Characteristic	Alive (*N* = 186)^a^	Dead (*N* = 63)^a^	Univariable analysis			Multivariable analysis		
			HR	95% CI	*p*‐value	HR	95% CI	*p*‐value
Sex					0.49			**0.036**
Female	79 (42%)	24 (38%)	—	—		—	—	
Male	107 (58%)	39 (62%)	1.19	0.72, 1.98		0.47	0.23, 0.96	
Age	45.70 ± 15.88	55.17 ± 16.23	1.04	1.02, 1.05	**<0.001**	1.02	1.00, 1.04	0.076
BMI					0.52			0.21
Normal	93 (50%)	32 (51%)	—	—		—	—	
Overweight	93 (50%)	31 (49%)	0.85	0.52, 1.40		0.68	0.37, 1.24	
Smoke					**0.010**			**0.005**
Non‐smoker	156 (84%)	41 (65%)	—	—		—	—	
Smoker	30 (16%)	22 (35%)	2.04	1.22, 3.43		3.19	1.47, 6.93	
Alcohol					**0.050**			0.51
No	176 (94.6%)	53 (84%)	—	—		—	—	
Yes	10 (5.4%)	10 (16%)	2.09	1.06, 4.11		0.72	0.27, 1.93	
Family history					0.94			0.087
No	180 (97%)	60 (95%)	—	—		—	—	
Yes	6 (3.2%)	3 (4.8%)	1.05	0.33, 3.34		0.33	0.08, 1.34	
Tumor location					0.10			0.21
Parotid glands	157 (84%)	47 (75%)	—	—		—	—	
Submandibular glands	26 (14%)	16 (25%)	1.67	0.95, 2.95		1.68	0.86, 3.29	
Sublingual glands	3 (1.6%)	0 (0%)	0.00	0.00, Inf		0.00	0.00, Inf	
Treatment					**<0.001**			**0.015**
No treatment	7 (3.8%)	9 (14%)	—	—		—	—	
Surgery only	93 (50%)	23 (37%)	0.18	0.08, 0.39		0.36	0.14, 0.95	
Radio/chemo/chemoradio	11 (5.9%)	16 (25%)	0.95	0.42, 2.16		1.07	0.36, 3.17	
Surgery+radio or chemo	75 (40%)	15 (24%)	0.14	0.06, 0.32		0.25	0.09, 0.71	
Facial nerve invasion					**0.011**			0.18
No	167 (90%)	50 (79%)	—	—		—	—	
Yes	19 (10%)	13 (21%)	2.39	1.29, 4.43		2.24	0.67, 7.51	
Capsule invasion					**0.010**			0.40
No	161 (87%)	47 (75%)	—	—		—	—	
Yes	25 (13%)	16 (25%)	2.24	1.26, 3.97		1.63	0.54, 4.88	
Skin invasion					**<0.001**			
No	171 (92%)	63 (100%)	—	—				
Yes	15 (8.1%)	0 (0%)	0.00	0.00, Inf				
Margin					0.31			0.78
Negative	169 (91%)	60 (95%)	—	—		—	—	
Positive	17 (9.1%)	3 (4.8%)	0.57	0.18, 1.83		0.81	0.19, 3.57	
Nerve cut					0.49			0.55
No	167 (90%)	59 (94%)	—	—		—	—	
Yes	19 (10%)	4 (6.3%)	0.71	0.26, 1.96		1.47	0.43, 5.07	
Size over 4 cm					**0.008**			0.59
No	151 (81%)	41 (65%)	—	—		—	—	
Yes	35 (19%)	22 (35%)	2.08	1.24, 3.50		1.23	0.58, 2.59	
T stage					**0.023**			0.95
T1/T2	86 (46%)	23 (37%)	—	—		—	—	
T3/T4	100 (54%)	40 (63%)	1.80	1.08, 3.02		1.03	0.50, 2.12	
N stage					**<0.001**			0.056
No	129 (69%)	27 (43%)	—	—		—	—	
Yes	57 (31%)	36 (57%)	2.84	1.73, 4.69		1.79	0.99, 3.26	
M stage					**<0.001**			0.21
No	184 (99%)	54 (86%)	—	—		—	—	
Yes	2 (1.1%)	9 (14%)	6.80	3.33, 13.9		1.91	0.70, 5.21	
Pathology					**<0.001**			**0.005**
Mucoepidermoid	48 (26%)	4 (6.3%)	—	—		—	—	
Lymphoepithelioma	35 (19%)	8 (13%)	2.51	0.75, 8.32		1.68	0.48, 5.82	
Adenoid cystic	30 (16%)	3 (4.8%)	1.33	0.30, 5.96		0.40	0.08, 2.05	
Others	73 (39%)	48 (76%)	6.38	2.30, 17.7		2.48	0.83, 7.44	

*Note*: Bold indicates statistical significant value (*p* < 0.05).

Abbreviations: CI, confidence interval; HR, hazards ratio.

^a^ *n* (%); mean ± SD.

In the whole cohort, 81.9% of the tumor site was the parotid glands, with submandibular glands 16.9%, and sublingual glands 1.2%. The male proportion was slightly higher than the female proportion, while the proportion of patients with normal or overweight BMI was exactly the same.

### 3.2 Risk factors of prognosis

The univariate Cox proportional hazards regression suggested that smoking and alcohol consumption was higher in patients with poor prognoses. Younger age and lower tumor burden including no neural invasion, no capsular invasion, no skin invasion, tumor size no more than 4 cm, and lower TNM staging were protective factors. Treatment discipline containing surgery showed significantly better prognosis, with the HR 0.18 (0.08, 0.39) for surgery only and 0.14 (0.06, 0.32) for surgery plus radio or chemotherapy compared to patients receiving no treatment. And the HR for patients received only radiotherapy, chemotherapy, or radiochemo therapy was 0.95 (0.42, 2.16). The result that the resection margin did not influence the prognosis was somewhat counterintuitive. And patients with mucoepidermoid carcinoma had a better prognosis than others.

Multivariable analysis showed male gender was a protective factor with HR 0.47 (0.23, 0.96) compared to the female gender. Besides, smoking and non‐mucoepidermoid pathology subtypes were risk factors. Patients benefited more from treatment discipline containing surgery, consistent with the findings in univariable analysis.

### 3.3 Prediction model based on random survival forest

**FIGURE 2:**
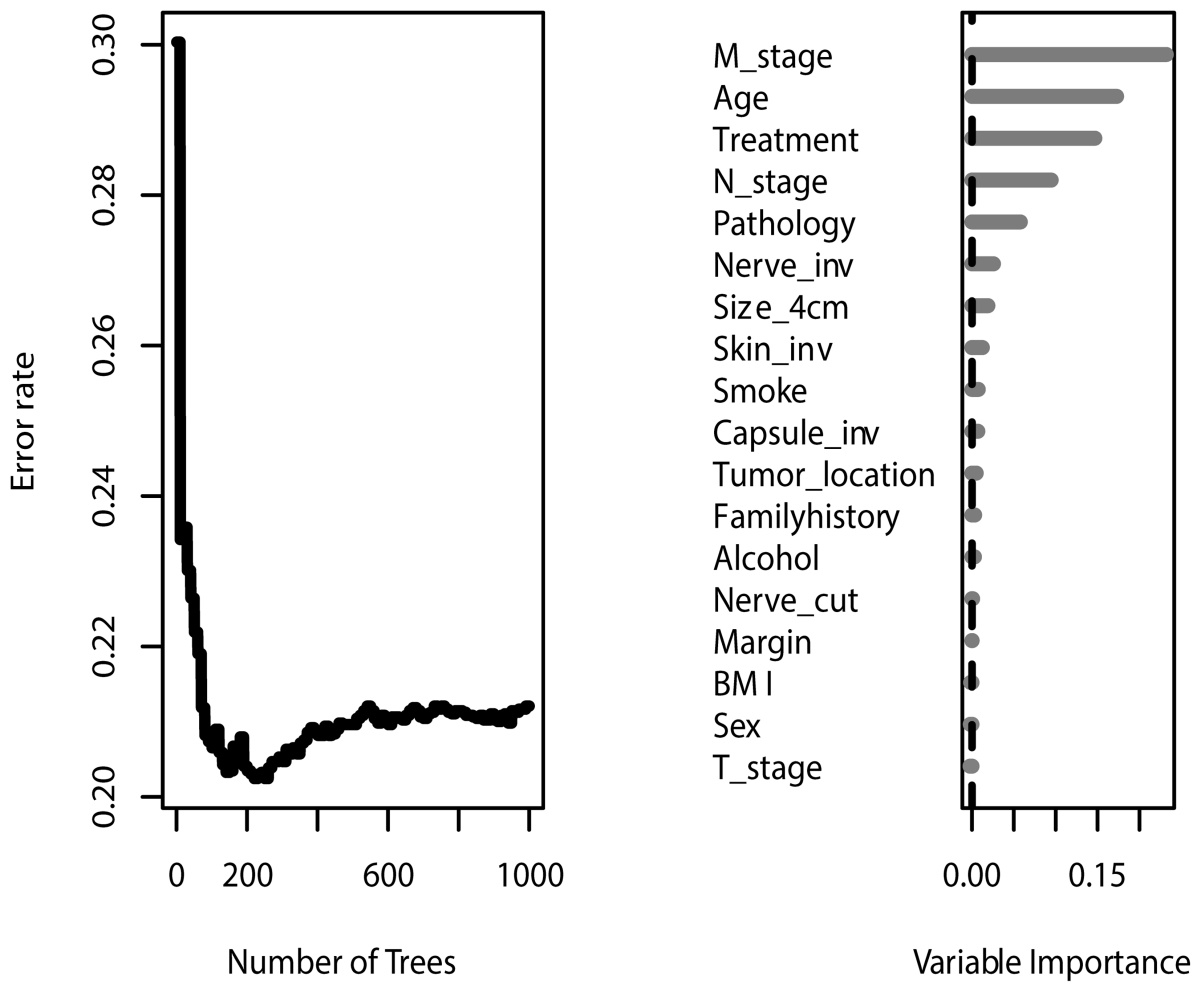
Results of the random survival forest.

**FIGURE 3:**
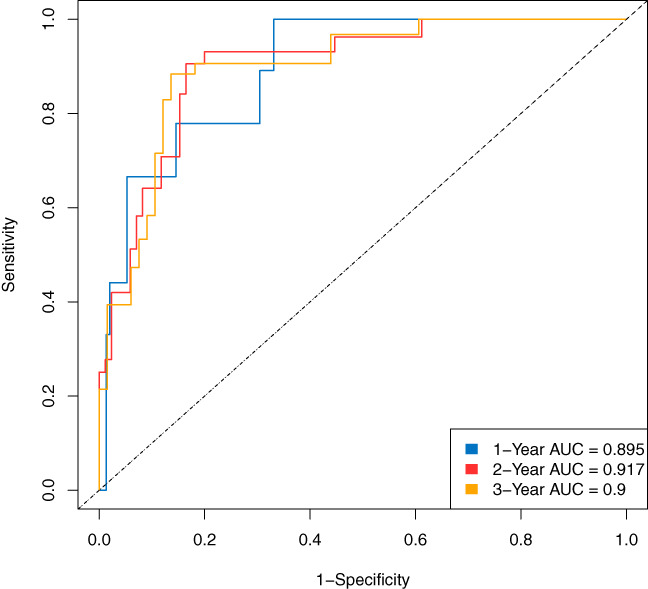
Time‐dependent receiver operator characteristic curve of random survival forest of training data.

**FIGURE 4:**
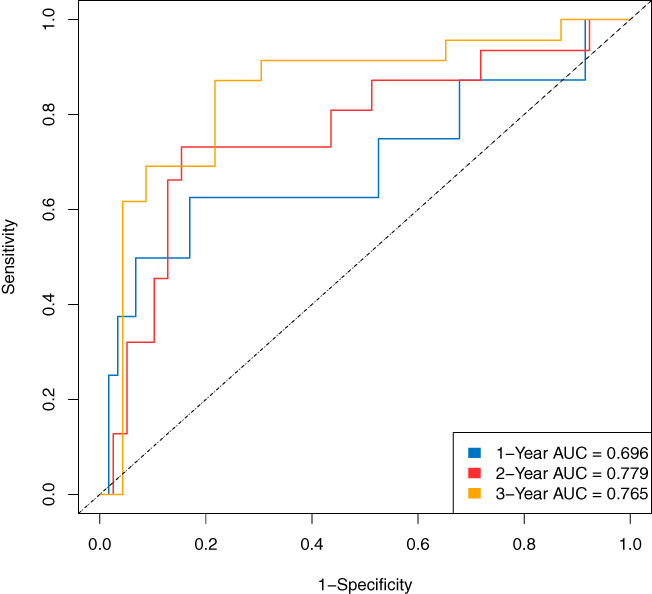
Time‐dependent receiver operator characteristic curve of random survival forest of validation data.

In the prediction model of the RSF, Figure 2 shows the variations of the prediction error rate along with the increasing number of trees and the importance of the variables. The top five variables that significantly influenced the model were M stage, age, treatment discipline, N stage, and the pathology type. This was in agreement with the Cox model analysis. The time‐dependent ROC showed the AUC of 1‐, 2‐, and 3‐year survival rates was 0.895, 0.917, and 0.9 respectively in the training data **(**Figure 3 **)**, and 0.696, 0.779, and 0.765 in the validation data **(**Figure 4 **)**, presenting a reasonable accuracy in the prediction of the OS, especially in the second and third year. We also built a prediction model based on the Cox model as a comparison, while the performance was not as good as the RSF (Figures S1 and S2).

**FIGURE 5:**
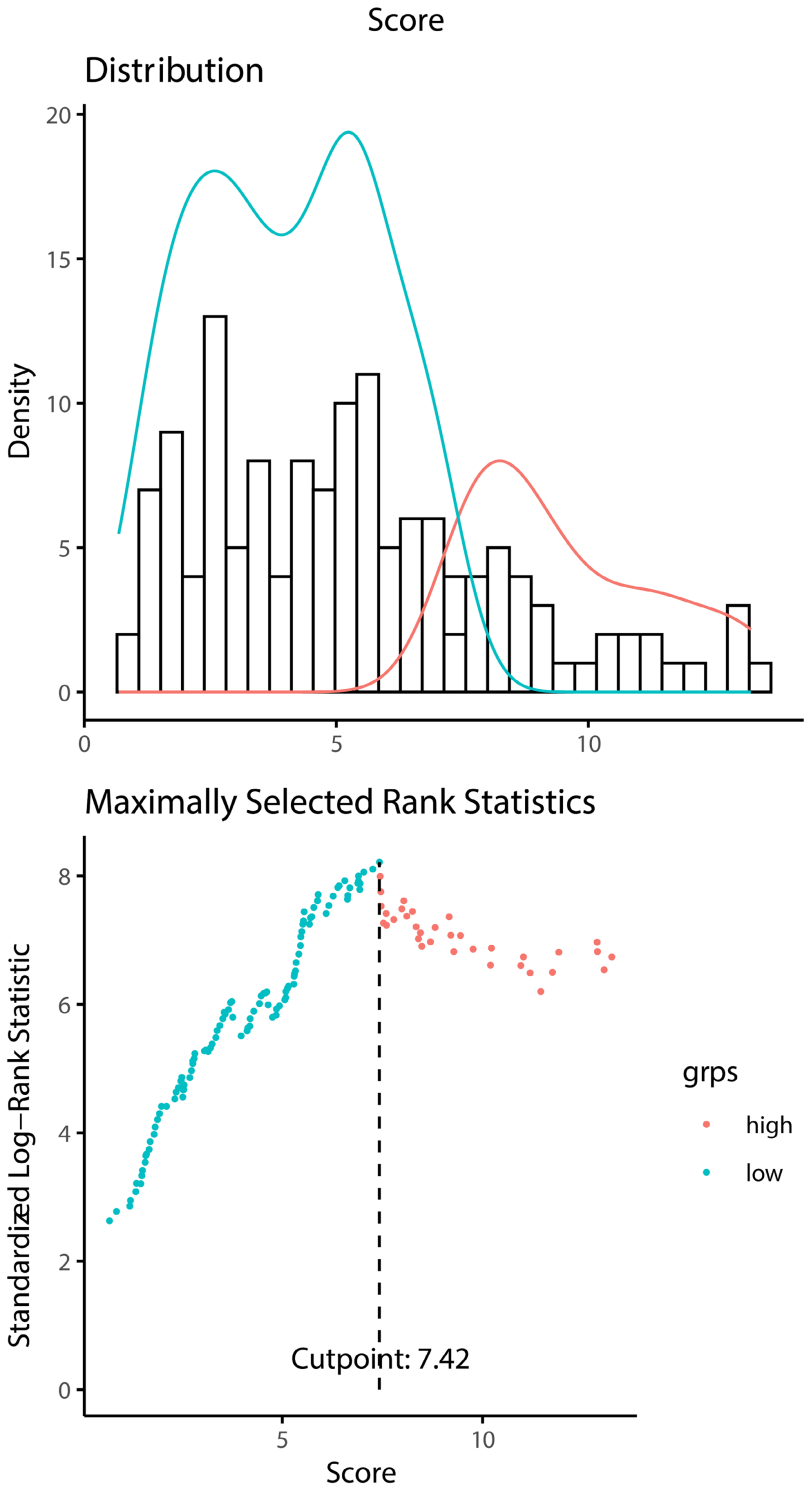
Cut‐off risk points calculated using the maximally selected rank statistics from the “maxstat” R package (an outcome‐oriented method providing a value of a cut point that corresponds to the most significant relationship with the survival).

**FIGURE 6:**
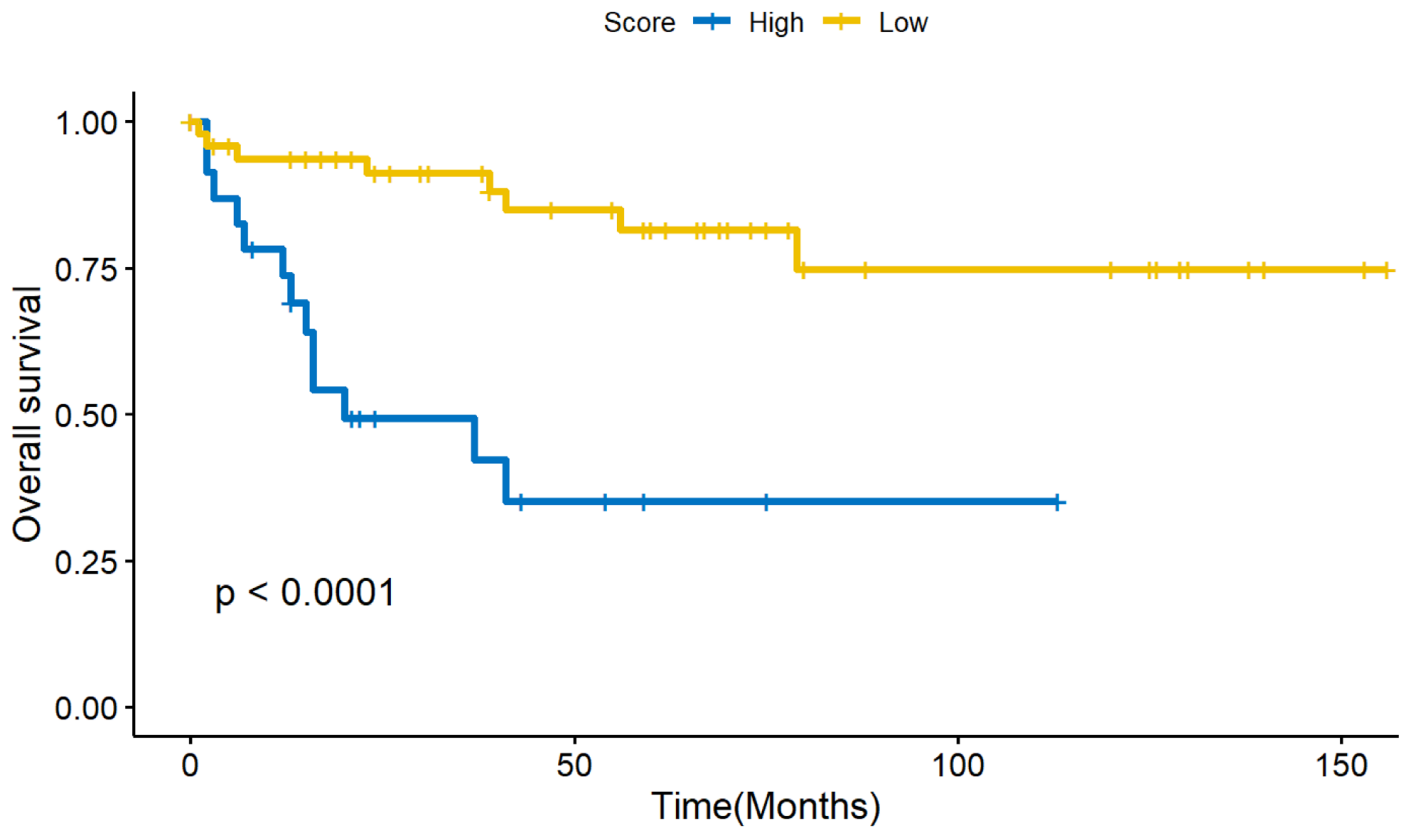
Log‐rank test of survival analysis between high‐ and low‐risk groups in validation data sets.

According to the RSF model, a risk score to indicate patient prognosis was calculated, presenting that the higher risk score was correlated with a worse prognosis. In the training data, the cut‐off risk score of 7.42 was selected using the maximally selected rank statistics from the “maxstat” R package, which is an outcome‐oriented method providing a value of a cut point that corresponds to the most significant relation with the survival (Figure 5). Then we used the cut‐off point and divided the patients into high‐risk group and low‐risk group in the validation data. Kaplan–Meier curves were performed for both groups, and the log‐rank test showed the OS was significantly different (Figure 6), proving that the model‐calculated risk score could effectively clarify patients with poor prognoses.

## 4 DISCUSSION

Improving the local control of the tumor and OS rate was the most crucial issue for doctors to consider. This study was performed to identify the factors associated with the outcomes and to develop a model to screen patients at high risk.

Age was reported as a significant factor for the OS in C‐MSG.^22^, ^23^, ^24^ We found the same results that younger age is a protective factor, which is the same among most head and neck cancer tumors, especially papillary thyroid tumors. Gender did not affect the prognosis in previous studies^23^, ^24^, ^25^ and is conflicting with our findings that the male factor suggested a protective factor in multivariable analysis. Possible explanations could be either bias due to the lesser size of the study or because we controlled more variables in the investigation, for the univariable analysis also showed no significant results.

Tumors greater than 4 cm have been reported to be related to a worse prognosis,^6^, ^24^, ^26^ which was consistent with our finding. The tumor size is usually an indicator of tumor load and is associated with the difficulty of complete resection of the specimen. Besides, we also found that nerve invasion, capsular invasion, and skin invasion could significantly influence the prognosis in the univariable analysis. The above results highlight the crucial role of surgery in the treatment discipline. And both the univariable and multivariable analyses indicated that patients receiving treatment containing surgery had a better prognosis. It suggested surgery is still the mainstay in the treatment of C‐MSG.

On the other hand, a counterintuitive finding was that the resection margin did not show a significant difference in the analysis. This discrepancy could be attributed to adjuvant radiotherapy. Chris reported postoperative radiotherapy (PORT) improved 10‐year local control significantly in patients with incomplete resection (82% vs. 44%),^27^ and the UK national multidisciplinary guidelines also recommended adjuvant radiotherapy should be considered in cases where there is incomplete or close resection margin.^28^ And a large‐scale study based on the National Cancer Database also reported that the positive resection margin indicated poor prognosis and PORT could improve the outcome significantly.^29^ However, the baseline characteristics in the above studies is unbalanced thus prospective evidence are still needed. Altogether, surgery plus adjuvant radiation and chemotherapy may be a better strategy for patients with advanced tumors, including capsular invasion, size over 4 cm, and incomplete or close resection.

The type of pathology also played an important role in our analysis. Patients with mucoepidermoid carcinoma presented with a better prognosis than the others not only in the univariable and multivariable Cox analyses but also in the RSF model. This is consistent with a previous study, which suggests low‐grade tumors have an indolent behavior and could be cured by surgery alone, whereas a high degree of malignancy tumors may be locoregionally aggressive and often require neck dissection and adjuvant therapy.^30^

The TNM staging system showed excellent capacity in evaluating the prognosis in the univariable analysis, and distant metastasis and lymph node status were also of great importance in the RSF model, broadly supporting similar findings by other researchers.^31^ However, in the multivariable analysis, no significant result was found. It may also likely be related to the discipline of surgery plus adjuvant radiotherapy, which greatly improved the prognosis in patients with advanced tumors.

The RSF model performed well in the validation data sets according to the time‐dependent ROC and was better than the Cox model, especially in the prediction of the second and third‐year survival. Besides, the RSF is like a “black box” and does not have to satisfy as many restrictions as the Cox model, thus is more applicable in clinical practice. The model calculates a score representing the risk of death over time for each patient so that the clinicians would clarify patients at high risk during a specific time, and a cost‐effective and individualized follow‐up strategy can be made. We believe this work would effectively guide our clinical practice to detect the recurrence in an early stage and take interventions in time. In addition, a more precise follow‐up could help save the limited medical resources, especially in a developing country with a large population like China.

Furthermore, to ease the use of the model, we calculated a cut point that could divide the patients with the most significant relation to survival. This helps us to determine whether a more aggressive therapy should be performed. For example, the positive resection margin is suggested to have a slight influence on survival in our analysis. Is adjuvant radiotherapy necessary for such patients in poor conditions? The RSF model may help us to make the decision.

On the other hand, chemotherapy is not routinely recommended in the treatment of salivary gland cancer, and just a few patients in our study accepted chemotherapy alone or adjuvant chemotherapy following surgery. Various regimens were chosen mainly based on particular pathological types (most were tumors metastatic to the salivary gland). We believe the small size of such patients is unrepresentative and plays little part in the analysis, thus we included them together into the same group to simplify the model.

The size and single center restrict further generalization of the study. Data from other centers would be necessary to improve the model and is the next direction of our work. The local recurrence may be more concerning to the clinicians, and we will try to track the outcomes of these patients. The RSF is not possibly appropriate for every clinical scenario, and indeed perfect model does not exist. But at least it works well and provides a promising method for us to screen patients with poor prognoses.

## 5 CONCLUSIONS

M stage, age, treatment discipline, N stage, and the pathology type significantly influenced the survival of the salivary gland tumor patients, and the prediction model based on the RSF could effectively screen patients at high risk of death.

## AUTHOR CONTRIBUTIONS

**Yufan Chen:** Conceptualization (lead); data curation (lead); formal analysis (lead); writing – original draft (lead). **Guoli Li:** Conceptualization (equal). **Wenmei Jiang:** Formal analysis (equal); validation (equal); writing – original draft (equal). **Rongcheng Nie:** Conceptualization (equal). **Honghao Deng:** Conceptualization (equal). **Yingle Chen:** Formal analysis (equal). **Hao Li:** Investigation (supporting); supervision (equal); writing – review and editing (equal). **Yanfeng Chen:** Conceptualization (lead); investigation (lead); methodology (lead); resources (lead); writing – review and editing (lead).

## FUNDING INFORMATION

This research did not receive any specific grant from the public, commercial, or not‐for‐profit funding agencies.

## CONFLICT OF INTEREST STATEMENT

The authors have no conflicts of interest to declare. None of the authors are current editors or editorial board members of Cancer Science.

## ETHICS STATEMENT

This study was in concordance with the Helsinki Declaration. The Institutional Review Board of Sun Yat‐sen University Cancer Center approved this study (approval number B2022‐221‐01).

## Supporting information


Figure S1

Figure S2

## Data Availability

The data that support the findings of this study are available on request from the corresponding author. The data are not publicly available due to privacy or ethical restrictions.
