# Supplementary figures and images for: Prognostic risk factor of major salivary gland carcinomas and survival prediction model based on random survival forests
Source: Cancer Med. 2023 Mar 19;12(9):10899–907. doi: 10.1002/cam4.5801 (PMC10225223; doi:10.1002/cam4.5801)

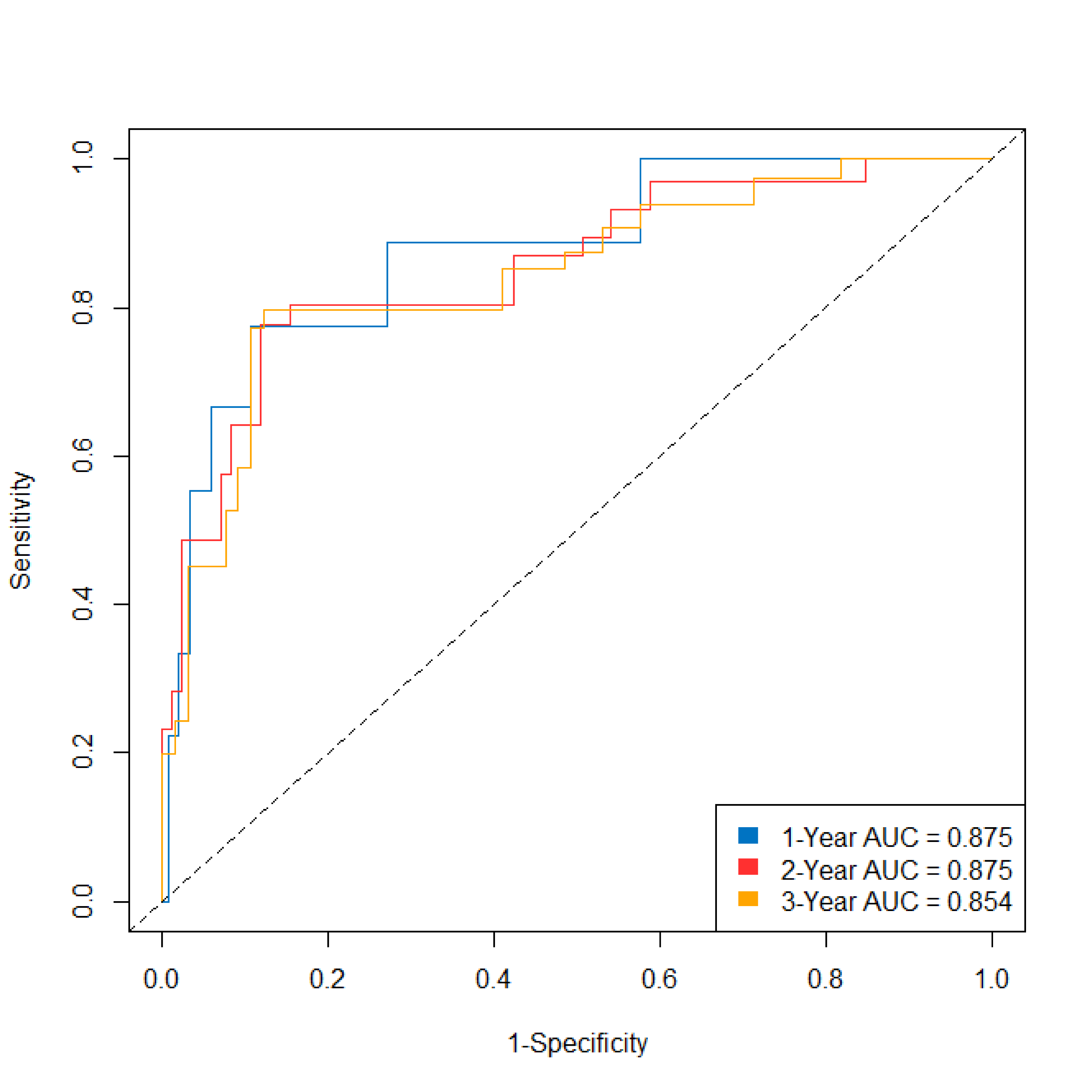

Supplement: Supplementary file 1 — Figure S1 [file CAM4-12-10899-s001.tif]

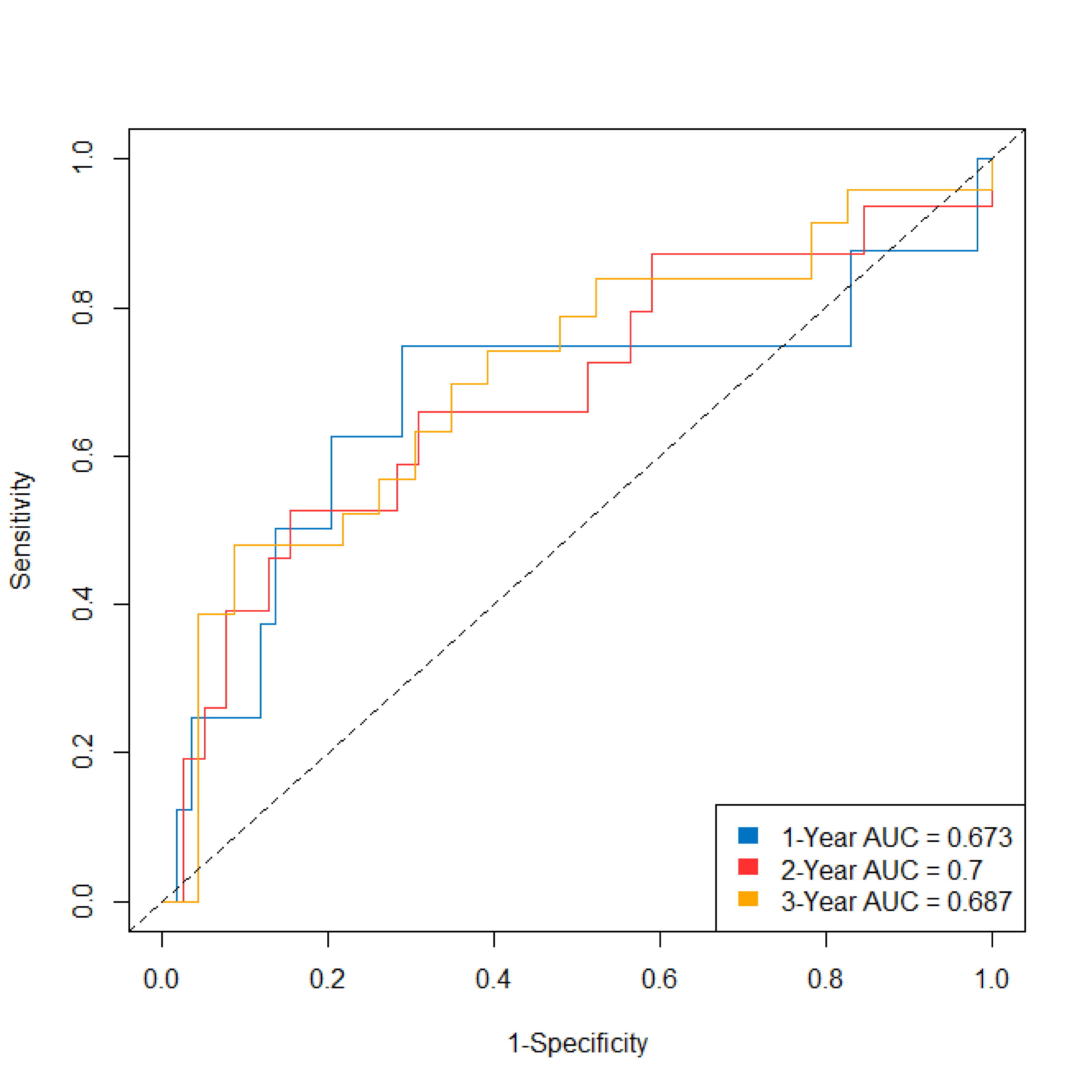

Supplement: Supplementary file 2 — Figure S2 [file CAM4-12-10899-s002.tif]
